# Comprehensive insights into health services accessibility and quality of life of families with individuals with 22q11.2 deletion syndrome in Brazil

**DOI:** 10.1186/s13023-024-03273-z

**Published:** 2024-07-06

**Authors:** Isabela Mayá Wayhs Silva, Vera Lúcia Gil-da-Silva-Lopes

**Affiliations:** Department of Translational Medicine, Medical Genetics and Genomic Medicine Area, Universidade Estadual de Campinas (Unicamp), Tessália Vieira de Camargo Street, 126, Campinas, Sao Paulo, SP 13083-887 Brazil

**Keywords:** Health service accessibility, Quality of life, Rare genetic disease, 22q11.2 deletion syndrome, Health equity

## Abstract

**Background:**

The 22q11.2 Deletion Syndrome (22q11.2 DS) presents unique healthcare challenges for affected individuals, families, and healthcare systems. Despite its rarity, 22q11.2 DS is the most common microdeletion syndrome in humans, emphasizing the need to understand and address the distinctive healthcare requirements of those affected. This paper examines the multifaceted issue of health service access and caregivers’ quality of life in the context of 22q11.2 DS in Brazil, a condition with diverse signs and symptoms requiring multidisciplinary care. This study employs a comprehensive approach to evaluate health service accessibility and the quality of life of caregivers of individuals with 22q11.2 DS. It utilizes a structured Survey and the WHOQOL-bref questionnaire for data collection.

**Results:**

Individuals with 22q11.2 DS continue to receive incomplete clinical management after obtaining the diagnosis, even in the face of socioeconomic status that enabled an average age of diagnosis that precedes that found in sample groups that are more representative of the Brazilian population (mean of 3.2 years *versus* 10 years, respectively). In turn, caring for individuals with 22q11.2 DS who face difficulty accessing health services impacts the quality of life associated with the caregivers' environment of residence.

**Conclusions:**

Results obtained help bridge the research gap in understanding how caring for individuals with multisystem clinical conditions such as 22q11.2 DS and difficulties in accessing health are intertwined with aspects of quality of life in Brazil. This research paves the way for more inclusive healthcare policies and interventions to enhance the quality of life for families affected by this syndrome.

**Supplementary Information:**

The online version contains supplementary material available at 10.1186/s13023-024-03273-z.

## Background

The intricate landscape of 22q11.2 Deletion Syndrome brings various challenges, impacting individuals, families, and healthcare systems. Despite being a rare genetic disease, with a global prevalence of 1 in 2148 livebirths, 22q11.2 DS is the most common microdeletion syndrome in humans [1–3]. This fact underscores the importance of understanding and addressing the unique healthcare needs of those impacted. One critical aspect of this challenge is the access of families affected by the syndrome to health services [4–6].

Spanning a diverse spectrum of signs and symptoms, 22q11.2 Deletion Syndrome can potentially involve almost every organ and body system [1, 7–9]. The most common clinical signs and symptoms include heart defects, palate abnormalities, immune system problems, developmental delays, hypoparathyroidism, and psychiatric issues. The complexity of 22q11.2 demands multidisciplinary and specialized care, including accurate diagnosis, appropriate treatment, and long-term support. In general, the evaluations recommended for adequate management over the different periods of development include pediatric/clinical, cardiac, nasopharyngeal, immunological, hematological, endocrinological, renal, audiological, ophthalmological, orthopedic, dental, psychiatric, gynecological, genetics and supportive therapies [10–12]. The interplay between the syndrome's complexities and the challenges of navigating health services influences the quality of life of people with 22q11.2 DS and their caregivers [6, 13, 14].

The access of individuals with rare genetic diseases in Brazil to the public health system is mainly through health centers and hospitals, generally not officially qualified to care for this population group [15–18]. The public health policy in force for specialized assistance to this public is Ordinance Nº 199/2014 also called National Policy for Comprehensive Care for People with Rare Diseases (Política Nacional de Atenção Integral às Pessoas com Doenças Raras - PNAIPDR). The Ordinance proposed that the care of individuals with rare diseases be comprehensive and coordinated between primary and specialized care to guarantee prevention, reception, diagnosis, treatment, and support until the resolution, follow-up, and rehabilitation of each case/patient. For this, rare diseases were grouped into 2 axes: axis 1, diseases of genetic origin, and axis 2, those of non-genetic origin.

The Ordinance Nº 199/2014 provides for 2 types of services: specialized care services for rare diseases (SCSRD) and referral services for rare diseases (RSRD) [19]. The main differences between both services are related to the number of diseases treated and the minimum number of professionals required for their qualification. For example, SCSRD does not require a geneticist as part of the minimum team.

The publication of Ordinance Nº. 199/2014 was a breakthrough for this population group. However, there are still critical gaps that prevent full access to health. These gaps permeate aspects such as disparities in the geographical distribution of services, a large proportion of authorized services not prepared to treat any rare diseases, absence of a geneticist in SCSRD, lack of integration between the Ordinance and the referral and counter-referral flows of patients, lack of data on rare diseases due to the non-obligation of registration and insufficient budget to cover the demand for diagnostic exams [17, 18, 20–22].

Regarding the access of individuals with rare genetic diseases to public health services through health centers and hospitals not officially qualified through Ordinance No. 199/2014, the lack of funds earmarked for the cost of diagnostic tests and limitations of knowledge about rare genetic diseases on the part of health professionals, generates, as a consequence, delay in diagnosis and clinical management and the non-referral of this individual to specialized services [17, 18]. In Brazil, the median age of 22q11.2 DS diagnosis is between 9.7 and 10 years of age [23–25].

Despite the growing recognition of the unique challenges faced by individuals with rare genetic diseases to access public health services in Brazil, there remains a significant research hiatus in understanding how these gaps impact the clinical management of specific syndromes such as 22q11.2 DS. This hiatus also includes knowledge about the quality of life of caregivers of individuals with 22q11.2 DS. In this way, this paper explores and analyses the multifaceted issue of health service access in Brazil within the context of 22q11.2 DS.

This article forms a crucial component of an extensive investigation study encompassing diverse approaches aimed at comprehending and assessing the health accessibility of families impacted by the 22q11.2 Deletion Syndrome (22q11.2 DS). The outcomes derived from this research were presented in 3 articles, each focusing on distinct aspects of the study. The first one reports the trajectory of families until diagnosis [24] (Part A). This paper is the second one (Part B), which delves into the evaluation of health services accessibility and the quality of life experienced by parents and guardians. The third article, entitle “Exploring Health Literacy in 22q11.2 Deletion Syndrome: A Comprehensive Study on Access to Information, Teleorientation, and Social Media Engagement in Brazil” (Part C), investigates health literacy accessibility among the affected families. Together, these articles contribute to a comprehensive understanding of the challenges faced by families dealing with 22q11.2 DS and offer valuable insights to guide future support and interventions.

This paper aims to help bridge these gaps by comprehensively examining access to health services and clinical management of this population and the quality of life of their caregivers. The findings of this study hold implications for policymakers, healthcare practitioners, patient advocacy groups, and affected individuals and families. By elucidating the access to health services of individuals with 22q11.2 DS and the quality of life of their caregivers, this research can potentially guide the development of more inclusive healthcare policies and interventions that can enhance the quality of life for those families affected by this syndrome.

## Methods

### Study design

This is a descriptive cross-sectional study. Data were collected through 2 surveys, one regarding access to health services and the other the caregiver’s quality of life, between August 2020 and May 2023. Eligible study participants were those who were parents or guardians of people diagnosed with 22q11.2 DS and were interested in participating. The exclusion criterion adopted was the absence of 22q11.2 Deletion Syndrome in the children or pupils. There were no age or biological sex restrictions.

Due to the absence of incidence data regarding 22q11.2 DS in Brazil and the challenges associated with accessing diagnostic tests, this study did not commence with a predefined recruitment target. Consequently, it takes on an exploratory nature, aiming to delve into the characteristics and challenges surrounding the syndrome within the Brazilian context.

Subject recruitment occurred through various channels, including WhatsApp groups of parents and guardians of individuals with 22q11.2 DS, @cienciaesaude.sd22q11.2 pages on Instagram and Facebook, sites of the Faculty of Medical Sciences of “Universidade Estadual de Campinas”, Brazilian Society of Medical Genetics and Genomics, as well as during meetings and lectures related to 22q11.2 DS. Both were made available online or through telephone calls. All participants signed the Free and Informed Consent Form (FICF).

The study was approved by the Ethics Committee of the “Universidade Estadual de Campinas” (Unicamp) (CAAE: 2477419.1.0000.5404) and was conducted according to the guidelines of the Declaration of Helsinki and Resolution no. 466/12 of the Brazilian National Health Council.

### Survey on access to health services

A structured Survey comprising 68 questions was designed based on current literature on the diagnosis and clinical management of 22q11.2 DS.

Based on the international recommendations of clinical management over the different developmental periods of an individual with 22q11.2 DS [10–12], the assessments available in the Survey to analyze the therapeutic itinerary after the diagnosis were: a) pediatric/clinical, b) cardiac, c) nasopharyngeal, d) immunological, e) endocrinological, f) renal, g) audiological, h) ophthalmological, i) orthopedic, j) psychiatric, k) genetic, l) hematological, m) gynecological and n) supportive evaluations. The assessment data shows that the individual performed 1 or more exams within that assessment category.

Individuals diagnosed with less than 1 year of age had their age of diagnosis classified as 0. For this study, regarding participants' education levels, individuals with incomplete primary education, complete primary education, and incomplete high education were classified into the primary education category. Those with complete high education and incomplete higher education were grouped under the high education category, while participants with completed higher education were categorized as having attained higher education.

### Caregiver’s quality of life

To assess the caregiver’s quality of life, we employed the structured questionnaire WHOQOL-bref. This tool, developed by the World Health Organization (WHO), has been validated in Portuguese and is widely used worldwide to assess quality of life, particularly in health-related contexts (FLECK, 2000; SEIDL; ZANNON, 2004).

The WHOQOL-bref comprises 26 questions, with 2 general quality of life questions and 24 questions representing four domains: environment, physical health, psychological well-being, and social relationships. Each domain's final scores were transformed into a scale ranging from zero to 100, where 100 signifies a higher quality of life.

The control group for comparative analysis of quality of life comprised parents of individuals without chronic disease.

### Number of participants

Not all participants who responded to the Survey on Access to Health Services also completed the quality of life questionnaire, and vice versa. Therefore, comparative analyses between quality of life and access to health services were conducted using the sample group that answered both questionnaires. Thus, in this article, there are 3 different tables of sociodemographic characterization: one for the Survey on Access to Health Services, one for the WHOQOL-bref, and one for those who answered both.

### Data analysis

Frequency tables of categorical variables were created, with absolute frequency (n) and percentage (%) values and position and dispersion measures for numeric variables.

When necessary, the chi-square or Fisher's exact test was used to compare categorical variables. To compare numerical measurements between 2 groups, the Mann-Whitney test was applied. The significance level adopted was *p* < 0.005.

## Results

### Survey on access to health services

#### Sociodemographic characteristics of the caregiver sample

In this approach, there were 65 caregiver participants, 61 mothers, 3 fathers, and 1 grandmother. Table 1 shows their sociodemographic characteristics. The majority of participants were women, aged 38, with higher education, and from the Southeast.

**Table 1:** Sociodemographic characterization of caregivers

**Characteristic**	***n* = 65**	
**Sex**	**F (%)**	**M (%)**
	62 (95.4)	3 (4.6)
**Age**	**Mean (s.d.)**	
	39.2 (7.8)	
	**Median (min-max)**	
	38.5 (24-65)	
**Maternal education level**	**Total (%)**	
Primary education	3 (7)	
High school	12 (27.9)	
Higher school	28 (65.1)	
**Father's level of education**	**Total (%)**	
Primary education	4 (9.5)	
High school	12 (28.6)	
Higher school	26 (61.9)	
**Region of residence**	**Total (%)**	
North	1 (1.5)	
Northeast	6 (9.2)	
Midwest	5 (7.7)	
Southeast	43 (66.2)	
South	10 (15.4)	
**Are you solely responsible for the individual with 22q11.2 DS?**	**Yes (%)**	**No (%)**
	22 (33.8)	43 (66.2)
**Are you currently employed?**	**Yes (%)**	**No (%)**
	33 (50.8)	32 (49.2)

*n* Number of participants, *F* Female, *M* Male, *s.d.* Standard deviation

Table 2 shows the sociodemographic characteristics of individuals with 22q11.2 DS related to the 65 participants in the Survey. The majority of them were men aged under 5 years.

**Table 2:** Sociodemographic characterization of individuals with 22q11.2 DS related to Survey participants

**Characteristic**	***n* = 65**	
**Sex**	**F (%)**	**M (%)**
	24 (37%)	41(63%)
**Age**	**Mean (s.d.)**	
	7.2 (7.1)	
	**Median (min-max)**	
	5 (0-35)	
**Age Group (in years)**	**Total (%)**	
0-1	4 (6.2)	
1-5	31 (47.7)	
6-12	18 (27.7)	
13-18	8 (12.3)	
> 18	4 (6.2)	

*n* Number of participants, *F* Female, *M* Male, *s.d.* Standard deviation

#### Access to health services

**Fig. 1:**
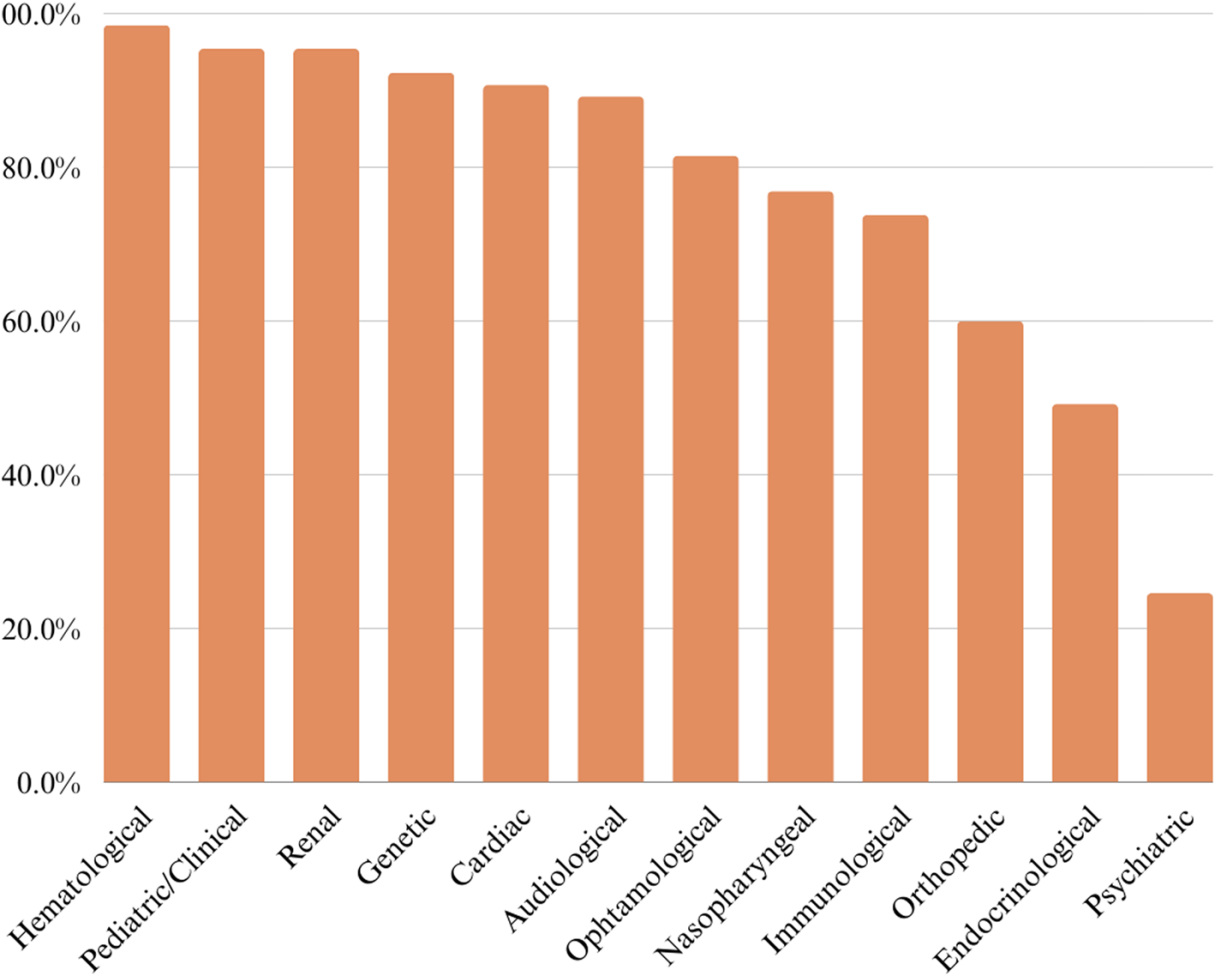
Access to expert assessment among the 65 individuals with 22q11.2 DS

**Fig. 2:**
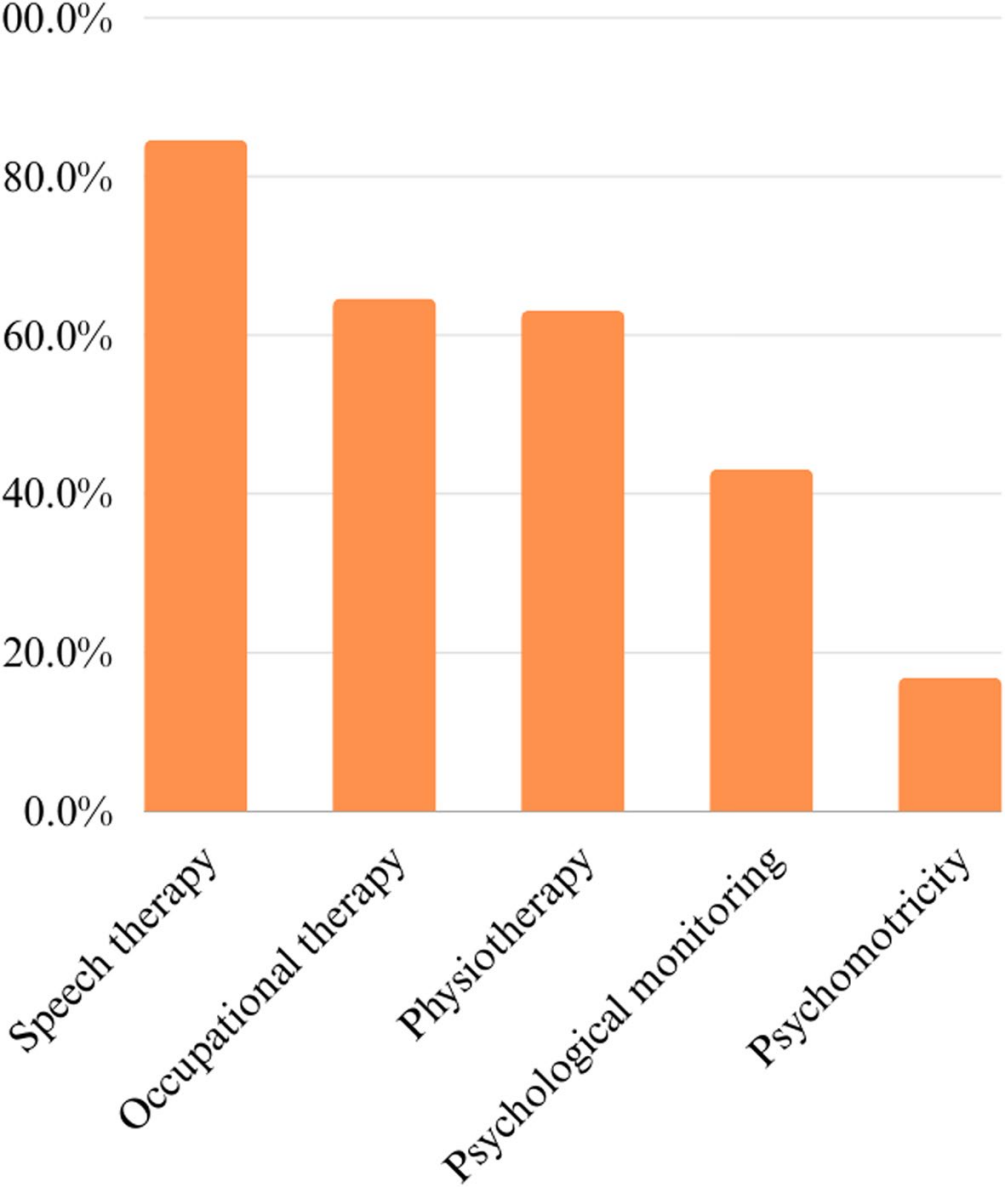
Access to supportive therapies among the 65 individuals with 22q11.2 DS

Among the 15 recommended assessments for proper management over the different developmental periods, 14 were included in the Survey. Data on access to gynecological evaluation and support therapies were analyzed separately. Tables 3, 4, 5, and Figs. 1 and 2 describe access to different health services. An additional file shows the tests used to confirm the 22q11.2 DS diagnosis [see Additional file 1]. Health Specialists were mostly accessed via private health services. The most frequently accessed assessments were hematological, pediatric/clinical, and renal. The least accessed was psychiatric evaluation.

**Table 3:** Access to health centers and professionals

**Characteristic**	***n* = 65**	
**Has regular follow-up in health services**	**Yes (%)**	**No (%)**
	64 (98.5)	1 (1.5)
**Travel to access health services**	**Yes (%)**	**No (%)**
	34 (52.3)	31 (47.7)
**Type of health service accessed**	**Total (%)**	
Health Center	7 (10.9)	
Health Center, Private Practice	14 (21.9)	
Health Center, Private Practice, First Aid	1 (1.6)	
Health Center, Private Practice, Emergency Room	1 (1.6)	
Health Center, Emergency Room	1 (1.6)	
Private Practice	37 (57.8)	
Private Practice, First Aid	2 (3.1)	
First Aid	1 (1.6)	
**Had difficulty getting care in any health specialty**	**Yes (%)**	**No (%)**
	35 (53.8)	30 (46.2)

*n* number of participants

**Table 4:** Access to 22q11.2 DS diagnosis

**Characteristic**	***n* = 65**	
**Age of suspicion of diagnosis**	**Mean (s.d.)**	**Median (min-max)**
	2.3 (4.3)	0 (0-25)
**Age of confirmation of diagnosis**	**Mean (s.d.)**	**Median (min-max)**
	3.2 (4.5)	2 (0-25)
**Age of access to the geneticist for the first time**	**Mean (s.d.)**	**Median (min-max)**
	2.8 (5.1)	1 (0-25)
**Parents who undergo genetic testing**		
Both parents	17 (26.2%)	
None of the two	41 (63.1%)	
Only mother	4 (6.2%)	
Only father	3 (4.6%)	

*n* number of participants

**Table 5:** Psychiatric disorders and access to treatment

**Characteristic**	***n* = 65**
**Diagnosis of psychiatric disorder**	**Total (%)**
	18 (27.7)
**Type of alteration**	**Total**
Anxiety	6
Anxiety, Depression	1
Anxiety, Depression, moderate mental retardation	1
Anxiety, Autistic Spectrum Disorder	1
Anxiety, ADHD	1
Depression	2
Neurocognitive Disorder	1
Schizophrenia and convulsive crisis	1
ADHD	2
Autistic Spectrum Disorder	4
Autistic Spectrum Disorder, ADHD	1
Moderate mental retardation	1
**Individuals with psychiatric disorder who access a psychiatrist**	**Total (%)**
	12 (66.7)
**Individuals with psychiatric disorder who access a psychologist**	**Total (%)**
	14 (77.8)

*n* Number of participants, *ADHD* Attention-deficit/hyperactivity disorder

Between the 12 recommended assessments evaluated together and available in the Survey, the average number of accesses per individual was 9.3 (s.d.=1.7, median = 9, min-max = 5-12).

Among the 6 female individuals aged over 10 years, 5 had already undergone a gynecological evaluation.

#### Number of professionals accessed versus the need to travel to access them

Individuals who reported the need to travel to access certain health professionals accessed more professionals compared to those who did not, corresponding to a mean of 9.44 ± 1.99 (median = 10, min-max = 5-12) *versus* 8.52 ± 1.52 (median = 9, min-max = 5-11) (*p* = 0.024, based on Mann-Whitney test) respectively.

#### Access to health services during COVID-19 pandemic

Among the 63 participants who answered the question: "Did the COVID-19 pandemic change access to consultations or therapies for individuals with 22q11.2 DS?" 43 (68.3%) said yes, it did, and 20 (31.7%) said no.

### Quality of life

#### Sociodemographic characteristics of the sample and control group

In total, 55 caregivers (52 mothers, 2 fathers, and 1 grandmother) and 61 controls (57 mothers and 4 fathers) answered the WHOQOL-bref. Table 6 shows their sociodemographic characteristics. Statistically significant differences have been found between the sample and control regarding maternal education, being the sole responsible for the child/adolescent, and being employed.

**Table 6:** Sociodemographic characterization of the sample and control group who answered WHOQOL-bref

**Characteristic**	**Sample (*n* = 55)**		**Control (*n* = 61)**		***p* value**
**Sex**	**F (%)**	**M (%)**	**F (%)**	**M (%)**	0.68^a^
	53 (96.4)	2 (3.6)	57 (93.4)	4 (6.6)	
**Age**	**Mean (s.d.)**		**Mean (s.d.)**		0.43^c^
	39.6 (7.8)		40.75 (6.7)		
	**Median (min-max)**		**Median (min-max)**		
	39 (24-65)		40 (29-56)		
**Maternal education level**	**Total (%)**		**Total (%)**		**0.007**^a^
Primary education	1 (3)		2 (3.3)		
High school	10 (30.3)		4 (6.6)		
Higher school	22 (66.7)		55 (90.2)		
**Father's level of education**	**Total (%)**		**Total (%)**		0.14^a^
Primary education	3 (9.4)		2 (3.3)		
High school	10 (31.3)		12 (19.7)		
Higher school	19 (59.4)		47 (77)		
**Region of residence**	**Total (%)**		**Total (%)**		0.09^a^
North	1 (1.9)		1 (1.6)		
Northeast	6 (11.3)		3 (4.9)		
Midwest	4 (7.5)		0		
Southeast	34 (64.2)		45 (73.8)		
South	8 (15.1)		12 (19.7)		
**Are you solely responsible for the individual with 22q11.2 DS (for control group: or for your children?)**	**Yes (%)**	**No (%)**	**Yes (%)**	**No (%)**	**0.0147**^b^
	17 (32.1)	36 (67.9)	8 (13.1)	53 (86.9)	
**Are you currently employed?**	**Yes (%)**	**No (%)**	**Yes (%)**	**No (%)**	**0.0009**^b^
	27 (50.9)	26 (49.1)	49 (80.3)	12 (19.7)	

*n* Number of participants, *F* Female, *M* Male, *s.d.* Standard deviation

^a^based on Fisher's exact test

^b^based on Chi-square test

^c^based on Mann Whitney test

#### WHOQOL-bref data

The scores obtained in the different WHOQOL-bref domains from both the sample and control group are described in Table 7.

**Table 7:** WHOQOL-bref general data from both sample and control group

**Characteristic**	**Sample (*n* = 55)**		**Control (*n* = 61)**		p* value
**Domain**	**Mean score from 0 to 100 (s.d)**	**Median (min-max)**	**Mean score from 0 to 100 (s.d)**	**Median (min-max)**	
Physical	60.97 (15.02)	64.29 (28.57-85.71)	72.66 (16.47)	78.57 (28.57-100)	**0.0001**
Psychological	62.80 (14.25)	62.50 (29.17-91.67)	67.96 (18.38)	75 (25-95.83)	**0.02**
Social relationships	60.15 (19.02)	66.67 (16.67-91.67)	61.20 (24.24)	66.67 (0-100)	0.65
Environment	61.12 (14.28)	59.38 (31.25-92.86)	70.14 (18.57)	71.43 (21.43-100)	**0.002**
QoL self-assessment	58.18 (26.38)	75 (0-100)	70.08 (24.07)	75 (25-100)	**0.02**

*s.d.* Standard deviation, *n* Number of participants

^*^Differences between sample/control were assessed with Mann Whitney test

### Access to health services and QoL

#### Sociodemographic characteristics of the sample

In total, 53 caregivers answered both the WHOQOL-bref and the Survey on access to health services, 50 mothers, 2 fathers, and 1 grandmother. Table 8 shows their sociodemographic characteristics.

**Table 8:** Sociodemographic characterization of the participants who answered both questionnaires

**Sex**	**F (%)**	**M (%)**
	51 (96.2)	2 (3.8)
**Age**	**Mean (s.d.)**	
	38.8 (9.5)	
	Median (min-max)	
	39 (24-65)	
**Maternal education level**	**Total (%)**	
Primary education	1 (3)	
High school	10 (30.3)	
Higher school	22 (66.7)	
**Father's level of education**	**Total (%)**	
Primary education	3 (9.4)	
High school	10 (31.3)	
Higher school	19 (59.4)	
**Region of residence**	**Total (%)**	
North	1 (1.9)	
Northeast	6 (11.3)	
Midwest	4 (7.5)	
Southeast	34 (64.1)	
South	8 (15.1)	

*F* Female, *M* Male, *s.d.* Standard deviation

#### Access to health services and QoL

Individuals who reported having difficulty getting care in some health specialty had a worse evaluation of the environment domain compared to those who had no difficulty, corresponding to a mean of 56.28 ± 11.95 (median = 56.70, min-max = 34.38-90.63) *versus* 65.68 ± 15.23 (median = 65.63, min-max = 31.25-92.86) (*p* = 0.024, based on Mann-Whitney test), respectively.

## Discussion

### Regional underrepresentation in study participants

Despite Brazil's population reaching 203.3 million [26] and the global prevalence estimate of 22q11.2 DS [27, 28], there is no specific data exists within Brazil. The engagement in the approaches herein presented under-represent the target population in terms of distribution across Brazilian regions. Most participants were females from the Southeast of Brazil with at least completed higher school education.

Regarding geographic distribution, 42.02% of the Brazilian population resides in the Southeast region, followed by 27.03% in the Northeast, 14.25% in the South, 8.86% in the North, and 7.83% in the Midwest [29]. However, the proportion of participants from the North and Northeast regions was much lower than expected, while the number from the Southeast region exceeded expectations. This fact is not associated with Internet access as in 2022 90% of households have access to the Internet in Brazil [30].

First, this limited representation in the North and Northeast regions can be attributed to the scarcity of reference centers for diagnosis. In the Southeastern states, each state boasts at least 1 reference service, whereas the North has only 1 reference service among its 7 states, and the Northeast, comprising 9 states, has reference services in only 4 [31]. Secondly, the concentration of medical geneticists is significantly lower in both the Northeast (14%) and the North (1.7%) compared to the Southeast (55.5%) [32]. The resulting limited access to specialized disease centers and medical geneticists in these regions may lead to delayed diagnosis and a lower proportion of diagnosed individuals compared to the Southeast [7, 24].

### Access to health services

The current initiative within the Brazilian public health system (Unified Health System - Sistema Único de Saúde - SUS) for comprehensive care for people with rare genetic diseases is Ordinance GM/MS nº 199/2014/PNAIPDR [19]. The overarching objective of PNAIPDR is to safeguard a continuum of care, spanning prevention, reception, diagnosis, treatment, sustained support until resolution, vigilant follow-up, and comprehensive rehabilitation for each case and patient. Crucially, the initiative fosters a seamless coordination between primary and specialized healthcare services. However, gaps must be overcome for this Ordinance to be fully implemented [17, 18].

Almost 100% of participants in the Health Survey reported that their relatives with 22q11.2DS access health services regularly, mainly through private health services. In Brazil, 71.5% of the population exclusively uses SUS, while 28.5% have some health plan [33]. Primary and secondary health services are primarily provided via the public system, while tertiary services are provided mainly by private services [34]. Studies indicate that individuals accessing private services have more success in obtaining care and a greater likelihood of using health services than those accessing public services [35, 36]. The recommended clinical management for individuals with 22q11.2 Deletion Syndrome primarily depends on secondary health services. However, diagnosis and genetic counseling are provided within tertiary public services, through the medical genetics specialty.

The research participants' educational attainment, coupled with their access to private healthcare services, suggests a socioeconomic status above the Brazilian population's average. Data from the Brazilian Institute of Geography and Statistics (IBGE) in 2022 revealed that just 53.2% of Brazilians had completed at least secondary education [37]. This higher socioeconomic status likely facilitated an average 22q11.2 DS diagnosis age in their family members of 3.2 years, contrasting sharply with the typical diagnosis age of around 10 years observed in two other studies encompassing different sample groups within Brazil [23–25].

Earlier access to diagnosis, in turn, did not translate to greater access to adequate genetic counseling, as 63.1% of the responses indicated that none of the parents had undergone a genetic test - a critical component for establishing risk and providing genetic counseling for the family [38].

Among the participants, 53.8% reported that their family members with 22q11.2DS had difficulty accessing medical specialties. Their family members accessed 77.5% of the Health Survey's recommended health assessments for clinical management. A previous study noted that individuals in Brazil with a mean age of diagnosis of 9.7 years had accessed only 68.75% of the recommended health professionals for their clinical management until the time of diagnosis, based on the assessments available in the Brazilian Database on Craniofacial Anomalies (BDCA) [23].

These data underscore the challenges of accessing the adequate clinical management despite the greater ease of accessing health services via private services. Among the potential causal factors contributing to inadequate clinical management, several key issues emerge: the scarcity of specialized health services across numerous Brazilian cities, compelling families to relocate to access a broader spectrum of healthcare professionals, as highlighted in the results section; the insufficient understanding of this syndrome and its treatment among healthcare professionals; and the lack of coordination between different tiers of care. It is essential to highlight that although there are already international management guides for 22q11.2 DS and an informal one designed in Brazil, there still needs to be an official 22q11.2 management protocol within PNAIPDR.

The health professionals least accessed in this study were psychiatrists and endocrinologists. Similarly, endocrinology was also the least accessed specialty in the health assessments up to the moment of diagnosis [24]. These findings are noteworthy, given that up to 60% of individuals with 22q11.2DS may present hypocalcemia due to parathyroid gland deficiency [12, 39].

Psychiatric disorders, including attention deficit, anxiety, impulsivity, autism spectrum disorders, and schizophrenia, are prevalent in up to 90% of individuals with 22q11.2DS, and initial signs are generally observable during childhood [12, 40–43]. The first access to a psychiatrist should ideally occur between 1 and 5 years of age, as early intervention can minimize and treat symptoms [11]. In this study, among individuals with 22q11.2 DS diagnosed with a psychiatric disorder, only 66.7% had seen a psychiatrist, and 77.8% had seen a psychologist.

### Quality of life among caregivers

Regarding the quality of life data, the results gleaned from this study underscore that caregivers of individuals with 22q11.2DS exhibit a lower quality of life across nearly all domains of the WHOQOL-bref questionnaire. This result aligns with other studies [44–49]. The social relationships domain represented the lowest score in our sample. Similar results have already been observed in caregivers of individuals with illnesses with multiple complications. They are linked to mothers being often solo caregivers and having little social support, which can impact the establishment of interpersonal relationships [50, 51]. In our study, 49.1% of caregivers for individuals with 22q11.2 DS were unemployed, with 32.1% of them being the sole carer. These results contrasted with those observed in the control group, where only 19.7% were unemployed, and 13.1% were solely responsible. However, the social relationships domain was also the one with the lowest score in the control group, which may be linked to cultural aspects and maternal burden in general, as the control group is composed primarily of mothers.

Further examination reveals that, when juxtaposed with each other, caregivers of individuals with 22q11.2DS who reported encountering difficulty accessing healthcare professionals for their family members exhibited a notably lower quality of life in the environmental domain than those who did not report such difficulties. The environmental domain is linked to feelings of security, finances, access to information and means of transport, and the physical environment of the residence [52]. This data emphasizes the interconnectedness of healthcare access challenges and the quality of life experienced by caregivers in specific domains.

Due to the complexity of 22q11.2 Deletion syndrome, caregiving for an individual with this diagnosis involves navigating numerous challenges. Caregivers' primary concerns typically emerge shortly after birth due to congenital changes that pose risks to the baby's life, such as cardiac changes, hypocalcemia, and palatal anomalies. Additional concerns arise after this period as other signs and symptoms become evident. Caregivers recurrently report anguish when contemplating the biopsychosocial insertion of their family member [6, 53, 54].

Furthermore, taking care of an individual with 22q11.2DS in the face of late access to diagnosis and inadequate clinical management is even more challenging. Therefore, addressing the multifaceted needs of caregivers, including improving healthcare accessibility and support systems, becomes imperative in the overarching goal of enhancing the overall well-being of individuals with 22q11.2DS and those caring for them.

### Recommendations

In light of the proposal of Ordinance No. 199/2014/PNAIPDR, which aspires to ensure the universality, comprehensiveness, and equity of health interventions tailored to the unique requirements of each rare disease, obtaining a contextual diagnosis of health access for diverse rare diseases within the ongoing PNAIPDR implementation becomes essential. This diagnostic endeavor is pivotal for crafting strategies that surmount existing gaps hindering the realization of its objectives in full measure. The official management guide for 22q11.2 DS must also be registered within PNAIPDR.

Regarding private health services, it is necessary to devise strategies to enhance health professionals' knowledge about 22q11.2 Deletion Syndrome. This initiative seeks to ensure that clinical management aligns with established recommendations and operates in a seamlessly coordinated manner.

### Limitations

One of the primary limitations of our study stems from the small sample size, which can be attributed to the rarity of the disease under investigation. Additionally, the ongoing pandemic likely contributed to difficulties in recruiting participants.

Also, adjustments were made to the sending format of the Survey and the WHOQOL-bref. Initially, they were sent together but in separate documents, including the FICF. However, after collecting responses only for the Survey or WHOQOL-bref, they were all combined into a single document, available for completion on the social networks created to publicize the project. This format did not allow the insertion of the confirmatory report of 22q11.2DS diagnosis for all participants. Therefore, confirmation of the diagnosis was based solely on answers to questions on this topic. Also, the participants reported the data obtained from memory, so there may be distortions concerning reality.

## Conclusion

Challenges in the North and Northeast regions possibly stem from a need for diagnostic reference centers and medical geneticists, leading to delayed diagnoses and undiagnosed individuals. While the current initiative, Ordinance GM/MS nº 199/2014/PNAIPDR, underscores a commitment to comprehensive care, gaps persist. The same can be said when it comes to private health services. The result is an inadequate clinical management of people with 22q11.2 DS. Quality of life data for caregivers reflects the broader impact of limited access to critical specialties, emphasizing the need for a holistic approach. Effective implementation of 22q11.2 DS requires concerted efforts to identify and address existing gaps and enhance healthcare accessibility and support systems. This study, part of a comprehensive and multifaceted investigation of SD22q11.2, contributes to revealing some important aspects in this direction.

### Supplementary Information

Additional file 1. Tests used to confirm the diagnosis. The additional file 1 provides information about the tests used by the 65 individuals to confirm the diagnosis of 22q11.2 Deletion Syndrome. 

## Data Availability

The datasets generated and analysed during the current study are not publicly available because it contains information from patients whose consent was given for specific use in this work.

## Abbreviations

22q11.2DS: 22q11.2 Deletion Syndrome
SCSRD: Specialized care services for rare diseases
RSRD: Referral services for rare diseases
Unicamp: Universidade Estadual de Campinas
WHO: World Health Organization
FICF: Free and Informed Consent Form
FISH: Fluorescence in situ hybridization
MLPA: Multiplex ligation-dependent probe amplification
NIPT: Non-invasive prenatal test
ADHD: Attention-deficit/hyperactivity disorder
SUS: Unified Health System
BDCA: Brazilian Database on Craniofacial Anomalies
